# Minimal Organizational Requirements for the Ascription of Animal Personality to Social Groups

**DOI:** 10.3389/fpsyg.2020.601937

**Published:** 2021-04-29

**Authors:** Hilton F. Japyassú, Lucia C. Neco, Nei Nunes-Neto

**Affiliations:** ^1^National Institute of Science and Technology in Interdisciplinary and Transdisciplinary Studies in Ecology and Evolution (INCT IN-TREE), Federal University of Bahia, Salvador, Brazil; ^2^Biology Institute, Federal University of Bahia, Salvador, Brazil; ^3^School of Humanities, University of Western Australia, Perth, WA, Australia; ^4^Faculty of Biological and Environmental Sciences, Federal University of Grande Dourados, Dourados, Brazil

**Keywords:** personality, organizational approach, social insects, group behavior, information, social minds

## Abstract

Recently, psychological phenomena have been expanded to new domains, crisscrossing boundaries of organizational levels, with the emergence of areas such as social personality and ecosystem learning. In this contribution, we analyze the ascription of an individual-based concept (personality) to the social level. Although justified boundary crossings can boost new approaches and applications, the indiscriminate misuse of concepts refrains the growth of scientific areas. The concept of social personality is based mainly on the detection of repeated group differences across a population, in a direct transposition of personality concepts from the individual to the social level. We show that this direct transposition is problematic for avowing the nonsensical ascription of personality even to simple electronic devices. To go beyond a metaphoric use of social personality, we apply the organizational approach to a review of social insect communication networks. Our conceptual analysis shows that socially self-organized systems, such as isolated ant trails and bee’s recruitment groups, are too simple to have social personality. The situation is more nuanced when measuring the collective choice between nest sites or foraging patches: some species show positive and negative feedbacks between two or more self-organized social structures so that these co-dependent structures are inter-related by second-order, social information systems, complying with a formal requirement for having social personality: the social closure of constraints. Other requirements include the decoupling between individual and social dynamics, and the self-regulation of collective decision processes. Social personality results to be sometimes a metaphorical transposition of a psychological concept to a social phenomenon. The application of this organizational approach to cases of learning ecosystems, or evolutionary learning, could help to ground theoretically the ascription of psychological properties to levels of analysis beyond the individual, up to meta-populations or ecological communities.

## Introduction

Do social animals constitute a new entity, a superorganism? If they do, this potentially independent new layer of organization could have a psychology of its own. This new social layer could then implement new social learning algorithms, potentially apart from the learning capacity of single bees or ants or any other social animal (Sasaki and Pratt, 2011). If this putative social psychology proves to be an emergent property in the hard sense, this new organizational level could have a personality of its own, even one that is different from the personality of the constituent individuals. Indeed, recent studies have evaluated at these upper organizational levels, from colonies to ecosystems, the onset of psychological phenomena that were originally described at the level of the individual, such as personality (Planas-Sitja et al., 2015), or learning (Power et al., 2015).

Before discussing the existence of these upper level, socio-psychological phenomena, such as social personality, one should be clear about what is precisely the lower-level phenomena, in this case, individual personality. Animal personality is defined as inter-individual differences in behavior that remain similarly different throughout repeated measurements performed in the same population (Carter et al., 2013). On the basis of this operational definition, personality has been attributed to highly unusual organisms such as anemones (Briffa and Greenaway, 2011) or even bacteria (Davidson and Surette, 2008). But operational definitions only specify, partially and temporarily, which kinds of operations count as empirical indicators for the referents of their concepts; they are temporarily in place of a concept that is the actual objective of the investigation, and they presuppose an underlying common cause for the measurements, without investigating the specific nature of the phenomena at stake (Feest, 2005), a situation that can potentially lead to puzzling outcomes, particularly when if this presupposition is violated. To evaluate more closely why the uncritical use of this simple operational definition of personality can be misleading, we propose a thought experiment.

Take the Homeostat, a simple electronic artifact basically constituted of four coupled control units (akin to batteries), a system that stabilizes the effects of any external disturbances introduced into it (Ashby, 1960). While a range of disturbances (inputs) to the Homeostat would result in the system returning to its stable configuration at a certain pace, Homeostats with slightly different initial configurations would return to stability at different paces, and this “inter-individual” difference in the pace to recovery would be stable and repeatable (Ashby, 1960). Stable and repeatable differences in the output (behavior) of individuals is the very operational definition of personality. Thus our thought experiment resulted in a puzzling outcome, i.e., the ascription of personality to a simple electronic artifact built in the 1960s. This rather absurd result emphasizes the difficulties that arise from the uncritical use of operational definitions.

While the use of simple operational definitions is certainly valuable when there is an agreement that the systems under analysis share basic organizational principles, like individual ants, bees, or spiders, the same should not necessarily hold when analyzing simultaneously systems at upper other levels of organization. Social organization does not need to mimic, and is not implied by, individual organization, and thus from the fact that a biological individual has personality (a lower level, intraindividual organization) it does not follow that a society or group of such individuals also should have a personality (at an upper level, social personality organization) of its own. Upper and lower levels of organization could adhere to distinct organizing principles, and if that is the case, operational definitions will not suffice. Thus, if we are investigating the presence of personality (or any other psychological phenomena) in upper-level biological systems, such as colonies, populations or ecosystems, the use of operational definitions could lead to unreliable outcomes, such as the one obtained above, in our Homeostat thought experiment.

A complete ontology of personality as a phenomenon is out of the scope of the present contribution, but it remains clear that personality is connected to basic defensive and approach information systems, in a general model of behavioral regulation (Corr, 2008, 2010). The regulation of behavioral action over the environment is a requirement for minimal autonomous agency (Moreno, 2018), and personality would be a particular and individualized way of regulating behavioral expression. Thus, a minimal definition of personality would consider it as a particular, individualized way of sensing and processing information, while stable behavioral outputs, repeatable across contexts, would be the outcome of personalities. This simple step allows a closer circumscription of the phenomena. If personality is a particular, individualized way to sense and process information, one could measure the information processing organization not only indirectly (through behavior), but also directly, evaluating the functioning of the circuitry underlying individualized behavioral control (Neubauer and Fink, 2010). This small definitional step in the direction of an ontology of personality suffices for our needs in the present study because, if personality is a particular and stable way to sense and process information, then social personality would also require a particular, underlying, stable organization, now at the social level, devoted for social information sensing and processing.

This definitional step leads to the discussion of the minimal requirements for autonomy in an organization devoted to information processing, and here we take advantage of the organizational approach in philosophy of biology to formalize these minimal requirements. For example, the organizational approach establishes the requirement of a regulatory feedback system that computes over the various subsystems, performing second-order closure of constraints (e.g., Moreno and Mossio, 2015).

Therefore, in this paper, we aim to analyze the adequacy of the application of social psychology terms to colonies, with the aid of Moreno and Mossio (2015) organizational approach to minimal autonomous agency, and using social insects as a case study. We will focus particularly on specific, well studied case systems of social insect behavior, case systems which provide enough detail for us to perform this organizational analysis. As a consequence, our analysis will apply to social insects in general only insofar as the behaviors (for example, foraging recruitment, house hunting) herein developed share general features across taxa systems. Throughout the paper we use the concept of social cognition as an emergent phenomena not reducible to individual cognition, one that is fundamentally based on the interactions between individuals, on participatory capabilities (De Jaegher et al., 2010).

To perform our analysis, we will start presenting an overview of the uses of social personality concepts in the literature. We will highlight the generalized ample use of an operational definition of animal social personality, and point to the need for a better understanding of its ontology, so as to adequately apply the concept of individual personality to a new, social level of organization. We will then summarize the use of social information networks in exemplar cases of social insects, searching for this new autonomous level of social organization. From that, we will present Moreno and Mossio (2015)’s organizational approach and the minimal requirements for the realization of cognition in autonomous systems. We shall move on then to the application of this organizational approach to examples of social insect colonies, analyzing the occurrence of social cognition on those systems. We end up concluding that socially self-organized systems, such as ant trails, or bee’s recruitment groups, are too simple to have social personality. We briefly extend our conclusions to discuss other similar cases, such as ascriptions of learning for whole ecosystems.

## Individual and Social Personality

Until a couple of decades ago, most of the behavioral differences among individuals of the same species were considered only slight variations due to plasticity or noise that could be dismissed in behavioral studies (Japyassú and Malange, 2014). Exceptions were the behavioral variation in different castes in social insects (e.g., Wilson, 1971) and the discrete alternative behavioral strategies in a few species (Gross, 1996; Widemo, 1998). The identification of consistent behavioral differences between individuals across contexts and/or time in a number of species resulted in the field of animal personality. In this field, inter-individual variation is recognized as a different source of behavioral variation that not only can be selected for, it can also persist and be transmitted through generations (Dingemanse and Réale, 2013).

Animal personality has been studied in invertebrates and vertebrates, in wild and domestic animals, with important ecological and evolutionary implications. An example of significant evolutionary implications of personalities is the effect of correlated behaviors in the evolution of a phenotypic trait (Sih et al., 2004). Considering that different behaviors are connected through the same personality type, they evolve as a package even when under contrasting selective pressures. Personality acts as a constraint for selection (Lynch and Walsh, 1998) and can result in apparently suboptimal characters (Dingemanse and Réale, 2013).

There are many factors thought to affect the evolution and maintenance of animal personality. They include genetic differences between individuals (e.g., van Oers et al., 2005), as well as physiological constraints that can change during the lifetime of an individual. Also, individuals’ experiences and environmental factors are extremely important. Specifically, social behavior seems to play an important part in the development of animal personality. The frequent interactions between individuals facilitate behavioral consistency and personality diversification in the colony, reducing the conflicts amongst its members (Bergmüller and Taborsky, 2010).

More recently, this concept is being applied to groups. From studies that identify consistent collective behavior in some species, colonies as a whole are considered to have personalities (for a recent review, see Wright et al., 2019). This is a result of the understanding that a colony is a reproductive unit and will be selected as a different level of organization (Jandt et al., 2014), developing their own behavioral characteristics. The definition of group personality is the same as the definition of individual personality (consistent behavioral differences across contexts and/or time), only that measured using characteristics of the collective, and not individual behavior (Wray et al., 2011; Scharf et al., 2012; Bengston and Dornhaus, 2014; Bengston and Jandt, 2014; Jandt et al., 2014; Blight et al., 2016; Jandt and Gordon, 2016; Pasquier and Grüter, 2016; Marting et al., 2018; Wright et al., 2019).

Similar to individual personality, group personality seems to be affected by factors such as genetics, physiology, and environment, but in the case of group personality, these factors are measured at the group level (Wright et al., 2019). This application is also possible because cohesive social groups can be taken as individuals, as in the case of social insects’ super organisms (Holldobler and Wilson, 2009).

Since collective personality requires collective behavior, it is important to understand how authors differentiate collective behavior from individual behavior. Collective behavior is considered to be an emergent property of the behavior of individual workers (Camazine et al., 2001; O’Donnell and Bulova, 2007; Sumpter, 2010). However, most collective personality studies do not explain the mechanisms involved in the behavior being tested, so that the ascription of collectivity is given by the nature of the test. If the test measures a collective outcome, such as colony defensive behavior, nest repair (Wray et al., 2011) or exploratory activity (Blight et al., 2016), the behavior would be considered collective. Here, one possible problem with these approaches it that collective behavior or decision can sometimes derive basically from individual behavior or decision (Huebner, 2013; Feinerman and Korman, 2017), and in these cases we would expect individual personalities to determine collective personality. When that is the case, the collective outcome could be explained merely by individual behavior; moreover, there would be no autonomous organization (in the sense of Mossio and Moreno, 2010; Moreno and Mossio, 2015) at the social level. In the absence of social autonomy there would be no reason to measure personality at both the lower (individual) and the upper (social) level, since one level predicts the other. One interesting example is the work of Jolles et al. (2017), that explains fish collective behavior through variations of two axes of individual personality (sociability and exploration). Their mechanistic model, based on individual personality traits, predicts the structure of the group, leadership and group foraging behavior.

Our review agrees with previous reviews (Jandt et al., 2014; Wright et al., 2019) showing the prevalence of a pragmatic, essentially operational definition of social personality, one that is based on the repeatability of (group) behavioral scores across contexts and time. Operational definitions describe how to identify the phenomena in the object of study, instead of defining exactly what personality really is. In other words, they tend to be much more descriptive (pointing to what there is), instead of proposing a concept or a theory, from a more prescriptive point of view. This is the reason why it is possible to apply an (operational) definition of personality to objects to which we would not intuitively ascribe personality, such as the Homeostat electronic artifact (Ashby, 1960, see section “Introduction”). This is another possible problem of directly upgrading to the social level operational definitions of personality that were conceived at the individual level: the scope of validity of the concept (for instance, evaluating the consequences of applying it to limiting cases) should be critically examined before the upgrading. This is definitely something that needs further investigation and clarification, a work appropriate for a conceptual—both scientific and philosophical—analysis.

In human studies, personality requires a coordinated and consistent response to environmental challenges. Accordingly, we would expect a minimum form of integration in the animal groups to be able to apply this concept at the social level of organization. Unfortunately, it is not clear, in most personality studies, if there is any mechanism that would be responsible for this integration, such as information sharing, or if the same results would obtain if each individual was acting independently, without social coordination.

In this paper, we aim to highlight one important characteristic that justifies the application of this concept to a group: there is a new level of organization and because of that, a new selective pressure. One way to identify this organization is through the flow of information within the system resulting in an autonomous social entity. In the next section, we summarize the use of social information networks in exemplar cases of social insects. Then, we will present an organizational approach to autonomous agency that can help us analyze the use of the concept of individual personality at the colony level.

## Information Networks Within Social Insects

Social insects are model animals in the study of social behavior, and communication pervades the organization of the colony, regulating relevant social tasks, from the recruitment of foragers (Dornhaus et al., 2003; Thom et al., 2007), to the selection of novel nest sites, or the organization of internal tasks within the colony (Seeley et al., 2012). It seems clear that there are information flows within the colony (Alem et al., 2016; Reznikova, 2017), and communication seems to be so central to social insect organization that the experimental disruption of relevant communication channels can even revert a social species to a solitary way of life (Yan et al., 2017).

Information flow can rely on diverse communication mechanisms such as physical interaction, pheromone use, auditory calling, vibrational signals, and trophallaxis. While some signals are unconditionally amplified by all receivers (i.e., signal transmission without social modulation), resulting in strong and almost instantaneous responses at the level of the colony, such as scent alarm triggering escape responses in ants (Jeanson and Deneubourg, 2009), most colony tasks are socially modulated at various degrees. As one example, an ant from a group that is collectively transporting a large food item may lose contact with the scent trail during the task; to avoid losing the correct direction, the group decides based on the transient amplification of individual-based knowledge: individuals who do not know where to push the load, follow the others, while those who know push in the right direction (Gelblum et al., 2015). There are various ways for social interactions to result in collective decisions, some relying heavily on individual decisions, with no social modulation, and others with varying levels of social modulation, up to the point that some decisions are only available at the group level, including emergent collective cognition, for example, during nest construction (Feinerman and Korman, 2017).

Here we will summarize a few of the best-studied signaling systems in social insects, focusing on two exemplar case systems: the collective choice of new nest sites (house hunting) and the collective choice of new foraging patches. Considering the huge diversity of social systems within either ants (Heinze et al., 2017; Reznikova, 2020) or bees (Wcislo and Fewell, 2017), our narrow focus here is to be taken as a first exploration in the application of organizational principles to the nascent field of animal social personality. Thus, our conclusions will generalize to social insects only insofar as the systems herein developed share relevant properties across social insects’ organizations, such as the reliance on social signaling and the formation of self-organized social structures.

### Collective House Hunting

House hunting has been well studied in ants of the genus *Temnothorax* and in the honeybee *Apis mellifera* (Marshall et al., 2009; Sasaki and Pratt, 2018). Ant scouts recruit others to a new nest by tandem running, slowly guiding the novice to the new site (Franks and Richardson, 2006), and the poorer is the new nest site, the longer they pause before recruiting new novices, resulting in lower rates of recruitment for the poorest sites (Mallon et al., 2001). Ants do not usually rely on scouts that have visited multiple candidate sites; instead, they rely on the competition between alternative recruitment groups. Colonies show a preference for adequately sized cavities with small entrances and low interior light levels, choosing in a few hours the best option available (Franks et al., 2003). When the number of scouts tandem running for one site reaches a threshold they switch to a faster recruitment strategy, transporting directly novice scouts instead of slowly guiding them to the new site (Franks et al., 2002). This new recruitment strategy boosts the favored option, that soon becomes the dominant option.

For honeybee swarms, the process is partially similar to the ant’s house searching algorithm, but the algorithm is implemented with different mechanisms. Instead of tandem runs, bees recruit novices with a waggle dance indicating quality, direction, and distance of the candidate nest site. Some scouts cease dancing, while others switch their allegiance to other candidate nest sites (Camazine et al., 1999; Seeley and Buhrman, 1999, 2001; Visscher and Camazine, 1999). Finally, and contrasting to the ant procedure, bees include not only positive, but also negative feedback loops in the search algorithm, performing stop signals against rival nest sites, and thus increasing the reliability of the decision process (Seeley et al., 2012). The colony-level decision results mainly from scouts spontaneously stopping to dance for less favorable sites, and from stop signals against competing sites, resulting in more new scouts being recruited to the best site. Eventually, a consensus is reached, with all recruits dancing to one single option, and leading the swarm to take off to the new site.

#### Collective Choice of Foraging Patches

One of the largely studied social decision mechanisms in ants is mass recruitment (Kolay et al., 2020; Reznikova, 2020). The decision between alternative foraging patches in mass recruitment results from the conditional amplification of individual scent signals during mass recruitment: the first finder marks the trail with pheromone in her way back to the nest, thus recruiting others to the foraging patch, but in many species, the recruited foragers also strengthen the first trail markings only in their way back to the nest, i.e., after evaluating by themselves the foraging patch (Beckers et al., 1992a; Mailleux et al., 2003). When there are alternative simultaneous trails, the differential amplification of one of the alternatives eventually leads to one single lasting trail. This differential amplification can occur either by the recruitment of a larger number of scent marking scouts, as a result of strongly marked trails eliciting disproportionately stronger responses than weakly marked trails (Sumpter and Beekman, 2003), or by each scout marking the preferred route with higher pheromone concentrations (Jaffe and Howse, 1979; Beckers et al., 1992b). Direct contact between recruiters informs about the food type (Le Breton and Fourcassié, 2004) and appear to convey information about the location of food sources (Reznikova and Ryabko, 2011), thus potentially informing the choice among trails. Down regulation of a trail occurs when recruits reduce pheromone deposition (Czaczkes et al., 2013) or use a no-entry pheromone over a trail (Robinson et al., 2005). Now for *Apis mellifera*, the distance from the food source to the hive is an important parameter in the dance signaling system: round dances inform about nearby, while the waggle dances inform about more distant resources. In any case, bees only dance after returning from highly profitable resources, and the nature and quality of these resources are informed directly through the floral scents stuck in the recruiter’s body and through regurgitating resources at the dance floor. The cognitive feats of an individual dancer include, among others, measuring the hive-resource distance through the optic flow in the journey back to the hive, evaluating (from the hive) the angle from the resource to the sun through polarized light, changing the coordinate system of this celestial angle to the vertical plane of the hive comb, and transducing the optical flow distance to the duration of the waggle run (Holldobler and Wilson, 2009). The success of the recruitment for a foraging patch increases with the dance floor vibration intensity (Sandeman et al., 1996), and with the frequency of the shaking signal: the worker climbs and shakes successive nestmates, thus bringing new workers to the dance floor (Seeley, 1995). Recruitment decreases with the frequency of multi-functional stop signals (Kirchner, 1993). Stop signals also promote cross-inhibition between competing foraging patches (Seeley et al., 2012) and increase the number of bees retrieving the food resources from the dance floor to the interior of the colony (Thom et al., 2003). Retrieving food from the dance floor to the interior of the colony can also be increased by the tremble dance signal (Seeley, 1995), whereby the signaler wanders irregularly about the combs shaking their bodies with their front legs held overhead, recruiting passing bees to nectar processing.

### The Organizational Approach to Autonomous Behavior

Since we are assuming the organizational approach in philosophy of biology as a theoretical landmark to interpret the ascription of personality and cognitive functions to social colonies, we will describe this approach in this section.

Organizational approaches have emerged in philosophy of biology in the 1990s and are becoming prominent along the last decades (e.g., Schlosser, 1998; Collier, 2006; Christensen and Bickhard, 2002; Delancey, 2006; Mossio et al., 2009). Biological systems, organized in a closure of constraints, are not only more complex, but also enable the potential increase of functional complexity, when compared to the simpler and qualitatively distinct self-organized systems (Moreno and Mossio, 2015, p. 18). One of the reasons for the prominence of organizational approaches is its philosophically coherent and integrative, as well as heuristically fruitful grounding of the teleological aspect of the functional ascriptions in biology (which we will explain below).

In order to present the organizational approach by Moreno and Mossio, let’s consider the functional relationship between a trait and the organism of which it is a part. More formally, according to this perspective, a trait *T* has a function in the organization *O* of a system *S* if and only if the following conditions, *Cn*, are satisfied:

*C*_1_: *T* exerts a constraint that contributes to the maintenance of the organization *O*.
*C*_2_: *T* is maintained under some constraints of *O*.
*C*_3_: *O* realizes closure (Moreno and Mossio, 2015, p. 73).

**FIGURE 1:**
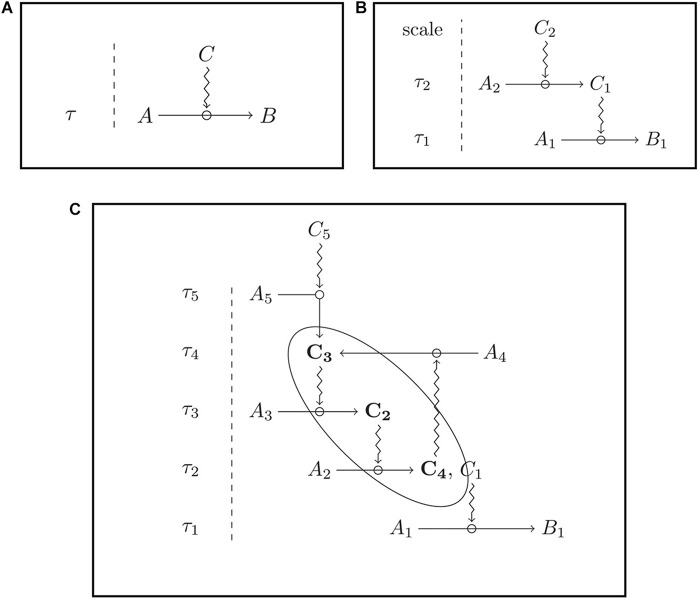
Constraints act upon processes and remain stable at the scale of these processual changes. **(A)** The constraint C acts over a process A > B; **(B)** dependence between constraints: the constraint C1 is dependent on the presence of another constraint, C2; **(C)** closure of constraints: the constraints C3, C2, and C4 are mutually dependent upon one another. Ai, Bi, and Ci are entities within a system; τi, specific time scales; the simple arrows indicate processes; the zig-zag arrow indicate constraining actions (from Moreno and Mossio, 2015, figures elaborated by Maël Montévil).

This definition can be illustrated with an example. On the one hand, the bee’s gut (*T*) exerts a constraining action on the physicochemical flow (represented by the ingested food) through all the bee body (the system *S*), and in this way contributes to the maintenance of the organization *O* of *S*. This corresponds to *C*_1_ in the formalization above, which represents a bottom-up influence (from the part to the whole system). On the other hand, according to *C*_2_, the gut (*T*) is maintained under constraints of the organization *O* of *S*. That is to say, the gut depends on other structures (such as the eyes, the wings, etc.) which constitute the very organization of the system *S*, since the gut needs, for instance, to receive nutrients (instead of toxic substances) that come from other organs, in order to maintain itself. This is a top-down relationship (from the organization as a whole to the part). Finally, according to *C*_3_ the organization *O* of *S* realizes closure, because of the very nature of the relationships described at *C*_1_ and *C_2_.* Closure, in general, means that a sequence of natural processes realizes a causal loop (see Nunes-Neto et al., 2014; Moreno and Mossio, 2015). For a schematic representation (see Figure 1).

There are two kinds of closure: closure of processes and closure of constraints. Closure of processes happens when, for instance, a process *A* causes a process *B*, which causes *C*, which, in turn, causes *A*. Some purely physical or chemical systems are characterized by a closure of processes. A closed glass bottle half full of water, receiving solar radiation is a good example. The solar radiation traverses the walls of the bottle and heats the water, which reaching one given temperature, evaporates. The water vapor rises and condensates in the top of the bottle, thus, falling as liquid water, which is now again subject to evaporation. The cycling of water molecules inside the bottle is a physicochemical circular flow, constrained only by external entities, in this case the glass and the solar radiation. The glass and the sun act, then, as external constraints, which are not regenerated by the cyclic thermodynamic flow of water.

By its turn, closure of constraints is a result of a complex organization, for which biological organisms are paradigmatic. A constraint happens when not only a flow of matter and energy (processes) forms a causal loop, but also biological structures (such as the bee organs) affect each other in mutual dependence relationships, and also determine a reduction in the degree of freedom of the flow of matter and energy, in other words, constrain the flow of matter and energy. Here, the idea of mutual dependence between constraints is crucial. Formally, a set of constraints *C* performs closure when, for each constraint *C*_*p*_, belonging to *C*, (i) *C*_*p*_ depends directly on at least one other constraint in *C* (i.e., *C*_*p*_ is *dependent*) and (ii) there is at least one other constraint *C*_*q*_, also belonging to *C*, which depends on *C*_*p*_ (i.e., *C*_*p*_ is an *enabling condition*). This mutual dependence generates the capacity of self-maintenance, which is specific to the way autonomous systems realize closure (for more details, see Moreno and Mossio, 2015).

In sum, going back to our example, we could say that the gut produces an effect (its function, to digest and absorb nutrients) which contributes to the maintenance of other organs (say, the wings), as it makes it possible that nutrients are delivered to them. The wings allow flying, which is a condition of possibility for finding new food, raw material for the gut performing its function, which closes the cycle.

As Mossio et al. referring to organizational closure, put it: organizational closure justifies explaining the existence of a process by referring to its effects: a process is subject to closure in a self-maintaining system when it contributes to the maintenance of some of the conditions required for its own existence. In this sense, organizational closure provides a naturalized grounding for a teleological dimension: to the question ‘Why does X exist in that class of systems?’, it is legitimate to answer ‘Because it does Y’ (Mossio et al., 2009, p. 825).

The organizational approach was originally applied to individual organisms and their traits as the functional units. However, we could conceive also the individual organisms, or the sets composed by them (such as colonies, populations, or communities), as the functional units themselves, thus applying the organizational approach to other levels within the ecological systems (Nunes-Neto et al., 2014, p. 131). More recently the scope of the functional units was broadened, in order to include abiotic items, once they can also play the role of constraints on the flow of matter and energy (El-Hani and Nunes-Neto, 2020). Thus the organizational approach can be applied to a broad range of levels in the biological hierarchy and, accordingly, we will use it to evaluate if one same psychological predicate can be found not only at the individual but also at the social level.

## Application of the Organizational Approach to Sociality

The organizational approach depicted above has a long history (see Gilbert and Sarkar, 2000; Wolfe, 2010), and allows for a principled ascription of functions within the biological hierarchy (Mossio et al., 2016), sanctioning functional ascriptions to both biological organisms (Mossio et al., 2009) and ecological systems (Nunes-Neto et al., 2014), a quality that is relevant for our purposes, since we are trying to specify the putative existence of a socio-psychological (a social mind) level on top of psychological individuals.

For example, the application of organizational principles to individuals has shown that to perform adaptively complex behavior, the cognitive system, as exemplified by the nervous system of individuals, should present some properties (Moreno and Mossio, 2015, pp. 167–193). First, as responsible for the mediation between internal (bodily) and external (environmental) sensorimotor coordinations, the cognitive system should not follow strictly the dynamics of either the internal or the external stimuli, that is, the cognitive system must show dynamic decoupling from the dynamics of both its internal (metabolic, physiologic) and external (ecologic) drivers. Second, the cognitive system should show second-order closure of constraints (see the section above), meaning that information should flow through a net of co-dependent constraints that are themselves generated within the very cognitive system. Third, for the possibility of adaptive adjustment in behavior, the cognitive system should be able to regulate its own functioning, meaning that there should be some internal constraints that only become active when the whole cognitive system is risking to fall out of bounds. The activation of these regulatory, constitutive constraints, could turn cognitive functioning back to normality either by maintaining the actual organization of constraints through calibrations on the flux of information, or by changing the actual organization of co-dependence between the constraints (i.e., the cognitive system enters into a novel organizational state). Together, these three requirements imply the autonomy of the cognitive system, meaning that the cognitive system is not merely responsive to either the internal (metabolic-physiologic) or the external (ecologic) drivers, but instead that it is an active system with its own structure and normative rules, built upon a history of interactions with these very drivers.

So, what happens when we jump from the cognition of an individual to the cognition of a social, or collective system? Social cognition in insects has sometimes been labeled as the product of a liquid brain (Solé et al., 2019), i.e., the product of a system where the “neurones” are not static (as in usual, solid brains) but instead are mobile agents (ants, bees, termites) that exchange, store and process information to obtain a collective decision. In this parlance, our question would thus be: do liquid brains have personality? To answer this question, we develop bellow a conceptual analysis, evaluating three requirements for autonomy of personality at a social level (Table 1 summarizes our main findings).

**TABLE 1:** Fulfillment of the organizational requirements for the ascription of social personality in particular cases of self-organized social processes.

Self-organized social structures	Social closure of constraints	Decoupling between individual and social dynamics	Regulation of collective decision processes	References
House hunting in Temnothorax	No	No	No	Mallon et al., 2001; Franks et al., 2002, 2003; Franks and Richardson, 2006
House hunting in *Apis mellifera*	Yes	No	No	Camazine et al., 1999; Seeley and Buhrman, 1999, 2001; Visscher and Camazine, 1999; Seeley et al., 2012
Recruitment for foraging in *Lasius niger*	No	Yes	No	Beckers et al., 1992a, b; Mailleux et al., 2003; Czaczkes et al., 2013
Decision between competing foraging recruitment trails in *Monomorium pharaonis*	Yes	Yes	No	Sumpter and Beekman, 2003; Robinson et al., 2005
Mass recruitment in *Atta cephalotes*	No	Yes	No	Jaffe and Howse, 1979
Decision between competing foraging recruitment vortices in *Apis mellifera*	Yes	No	No	Kirchner, 1993; Seeley, 1995; Holldobler and Wilson, 2009; Seeley et al., 2012
Integration between distinct tasks in *Apis mellifera*	Yes	No	No	Seeley, 1995; Thom et al., 2003

### Closure of Constraints at the Social Level

Information flow within the social system occurs through communication between individuals, and following our review of social insect communication, we find that these requirements for social cognition sometimes do not hold at the social level.

For example, the recruitment processes for the choice of a foraging patch, or of a new nest site, are paradigmatic examples of self-organized systems that constrain the flow of information to, and within the colony. But self-organization by itself does not imply the existence of a closure of constraints, because self-organized systems have one single, macro-level constraint, and therefore they are not able to instantiate any co-dependence between constraints (Mossio et al., 2009). In our exemplar case, although one single mass recruitment trail certainly constrains the flux of information to individual ants, feeding back the recruitment of new foragers to the trail, and thus contributing to its own self-maintenance (i.e., it is a self-organized system), it is constituted by one single constraint (the trail itself), and thus cannot by itself realize a closure of constraints. The trail is, in this organizational analysis, comparable to physicochemical dissipative structures, self-organized systems such as the flame of a candle, or Bénard cells (the bubbles that appear spontaneously when heating water), but it could not by itself be considered, at the collective level, a cognitive system.

But there is more to insect societies than isolated self-organized social structures (SOSSs). The collective choice between competing foraging patches (or competing nest sites) relies basically on the differential recruitment of new individuals to one of the competing options, through the positive feedback within each option, and also through the addition of negative feedback across competing options. Thus, when there are two alternative vortices of recruitment at the same time, we do have two collective structures constraining the flow of information through the individuals, so that there is the possibility of a co-dependence between constraints, and thus a possibility of fulfilling one of the requirements for cognition at the social level.

When the choice between competing recruitment options depends overly on the differential amplification of distinct trails, there is scant need for interactions between the two competing trails. When this is the case, the two trails are each one a constraint to information flow within the colony, but the constraints do not interact significantly with one another, there is no clear flow of information between the trails, meaning, again, that there is no closure of constraints. Thus, when there is no significant interaction between the constraints (no closure of constraints), the resulting phenomenon, the collective choice of one of the available resource patches, is fundamentally a result of individual cognition guided by self-organized processes. While this is certainly a social decision process, it does not reach the complexity required for a closure of constraints, and thus it does not attain autonomy at the social level. The competing trails would, in cases like this, be akin to distinct bubbles of water (Bénard cells) differentially growing through the “recruitment” of nearby water molecules during the heating of water, a recruitment that feeds back the competing self-organized processes.

**FIGURE 2:**
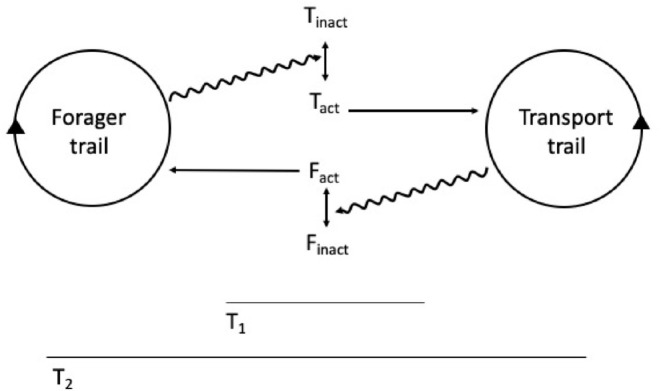
Forager and transporter trails (socially self-organized systems—SSSs) constrain information flow, thus causing individuals to change their internal state, between inactivity (inact) and activity (act). As a result, both SSSs act indirectly upon each other through recruitment processes. Both transporters (T) and foragers (F) are depicted; T1 and T2 are different time scales.

The situation seems different for *Apis mellifera* or *M. faraonis*, which show a significant interaction between distinct recruitment groups through stop signals that promote cross-inhibition between competing options (Robinson et al., 2005; Seeley et al., 2012). These species seem thus to rely on more than self-organization to choose the best option: there is signal processing between the competing vortices of recruitment, and thus the final choice involves a second order, across sites information processing system. This second-order information processing system could qualify as cognition at the social level, that is, on top of individual cognition because, from an organizational standpoint, when there is significant interaction between the constraints (recruitment vortices), there is the possibility of appearing a closure of constraints (see Mossio et al., 2009 and the section above). Interaction between SOSSs (the recruitment vortices in the example above) can also occur between distinct, spatially contiguous tasks in a colony (Figure 2). For example, while an external ant trail focus on bringing pieces of leaves into the nest, another trail focus on transporting these pieces to inner parts of the colony, or while a group of external forager bees focuses on bringing nectar to the comb, another group focuses on further nectar processing, within the colony. In these cases we also can have interactions between two self-organized activities: external forager bees make the tremble dance signal, whereby the signaler recruits passing bees to the internal nectar processing task (Seeley, 1995). There can also be indirect interaction between contiguous tasks: the continuous action of external ant foragers generates a pile of resources, which then stimulates workers within the colony to further process the pile, a process denominated stigmergy (Theraulaz and Bonabeau, 1999). Through either direct or indirect interaction, the complementary tasks (i.e., the distinct transport SOSSs) constrain the flow of information, and thus the decision process of individuals, in a way that instantiates a co-dependence between these very constraints (Figure 1).

The argument above could be more general, including not only two, but all the task forces in a colony, in species where there is heterogeneity of tasks. In general, considering the whole colony, heterogeneous interaction profiles across individuals emerge with colony size increase and, with that, information flow becomes modularized (Naug, 2009). The evolution of task specialization with colony size increase would thus create informational compartments within the colony, with highly connected individuals at the edge of these compartments (O’Donnell and Bulova, 2007). If direct or indirect connectivity between contiguous tasks imply closure of constraints, as discussed above, then the colony could be considered to have a second order, social information processing system.

### Decoupling Between Social and Individual Dynamics

Closure of constraints at the social level is but one of the requisites for autonomous social cognition. There must also exist a decoupling between the dynamics of the cognitive system and the dynamics of both its internal and external drivers, that is, in the case of individual cognitive agents, internal metabolic signaling (physiology) and external environmental stimuli. At the social levels, the equivalent to internal metabolism would be the social physiology, the decentralized interactions between colony members. But these interactions are not easily distinguishable from the information flow (between and within its SOSSs). This is because, differently from what happens within the nervous system, where the rapid dynamics of information flow surpasses the slower dynamics of body metabolism, thus allowing for an effective integration across distant body parts, at the social level, the information flow, both between and within SOSS, is obtained by these very interactions: information flow and interactions are one same thing. The second-order, social information processing system seems stuck in the same dynamics of the interactions between individuals (it is constituted by these very interactions), i.e., it is stuck in the dynamics of social physiology. This could explain why frequently the dynamics of collective behaviors mimics the dynamics of ecological drivers (Gordon, 2019): there is no autonomous level of social information processing, resulting in a social system that is by default coupled to its external drivers.

In some circumstances, however, the dynamics of social information could be decoupled from social interactions. This could occur when there is indirect interaction, through contact pheromones, or in the case of stigmergy. This is because indirect interaction relies on social assets (a collective mass of pheromones in a trail, a pile of resources), which have a dynamics that is slower than the dynamics of direct, inter-individual interactions. But while the second-order, nervous system based individual cognition is able to integrate distant parts of the organism because of its fast dynamics, social insects could sometimes have a second-order, social information processing system that is, on the contrary, slower than the direct interactions themselves. Slower processes cannot integrate a bunch of faster processes, and thus are unable to produce real-time, concerted social responses that attend simultaneously to various colony demands.

### Regulation of Collective Decision Processes

The last formal requirement for autonomy at the social level is the possibility of self-regulation of the social decision processes. This would require one or a few SOSSs that would enter into action when the system is out of bounds, interfering with information flow or with the very organization of constraints (Moreno and Mossio, 2015).

Although the existence of these regulatory constraints is possible, the very decentralized nature of colony organization, relying heavily on anonymous agents using local information and indirect interactions (Feinerman and Korman, 2017), seems to reduce the possibility of regulation through supplementary social structures. There is certainly regulation of interactions by individuals (Kolay et al., 2020). For example, *Monomorium* ants produce a volatile pheromone that repels workers from unprofitable resources (Robinson et al., 2005), thus contributing, for example, to the decline of an established trail. But, as we have discussed above, the whole trail (from its creation to its extinction) is a SOSS and, as such, it is a very intricate process from the point of view of the individuals that create it, but from the point of view of the social organization, it is way too simple. Any SOSS is constituted by one single constraint (see above “Closure of constraints at the social level”), and to allow for regulation one needs at least three constitutive constraints (SOSSs): a regulator constraint that enters into action only when needed (when the system is out of bounds), so as to modify the interaction between the two remaining, co-dependent constraints. We should be careful not to mix levels of analysis: regulation of individual interactions is paramount for an analysis at the level of the individuals, but we are here working at the social level, searching for SOSSs that regulate the interactions between other SOSSs.

## Discussion

Although we concur that the ascription of psychological predicates to individual ants, bees, or any other social animal, is itself literally correct (Figdor, 2018), the same may not hold true for the ascription of psychological predicates to upper, social level entities. Considering personality as connected to a very general view of cognition as information processing (Shettleworth, 2010), framing this general view within an organizational approach, a very effective theoretical development for studying functions across levels of biological organization (Mossio et al., 2016), we found conflicting results concerning the ascription of personality to social entities.

A general result of the conceptual analysis is that one cannot uncritically ascribe psychological predicates to self-organized social structures (SOSSs), such as termite or ant trails, or bee scouts recruiting for a single resource patch, because these social entities constitute themselves in a single constraint (to information flux). In this case, there is no upper individual, social level of cognition, no two self-organized social structures that generate one another and perpetuate themselves, by driving information flow through the colony.

To be clear, this is not to say that there is no emergent cognition. Self-organized processes are paradigmatic models for emergent phenomena, and cognition is no exception at it, for collective behaviors, such as collective motion patterns or collective predator avoidance patterns, do emerge from individual interactions (Ioannou et al., 2011). Individuals do communicate or interact with one another in these self-organized, social processes, but the collective, sometimes emergent outcome relies mostly on individual cognitions trapped in non-linear feedback loops that are characteristic of selforganized processes. The social structure that emerges in these cases is, from a modeling perspective, akin to a physicochemical dissipative structure, and in this way, it is too simple as a social structure, one that is unable to fit even in the simplest models of closure of constraints (Figure 1C). There is emergent cognition, but not one complex enough to be sustainable, autonomous at the social level: there is no social cognition.

This first conclusion has many practical consequences for research in social personality. For example, the finding of stable, across-colonies differences in a collective trail property, such as the timing to form mass recruitment trails, or the timing to recover from an experimental perturbation performed on such an isolated trail, the stability on any of these measurements should not be taken as an index of the existence of social personality, because they simply reflect a combination, albeit a non-linear and sometimes complicated one, of the individual personalities already present in that trail. No further psychological, autonomous social system of information processing is required to explain the observed pattern.

When the measurement of social personality involves not one, but two or more interacting trails, recruitment processes, or more generally, SOSSs, then our conceptual analysis portrays a more nuanced outcome. If there are negative and positive feedbacks occurring between the SOSSs (Figure 2, Table 1) the social system is complex enough to show closure of constraints, presenting a second-order, social information processing system on top of the first-order, individual information processing system. At a lower level of analysis, at the level of individual cognition, a second-order processing could be implied in the cross inhibition between integrating populations of neurons, and it would be crucial for effective behavioral choice in individual decision-making tasks (Bogacz et al., 2006; Bogacz, 2007).

But not all collective SOSSs choices (between food patches, nest sites, routes) possess a second-order, social information processing system: some choices, as the above discussed case of the Pharaoh’s ant, are better characterized as effected through a population of independent, barely interacting socially self-organized structures (trails). In these cases, the observation of any stable, cross-colonies differences in any of the collective choice’s attributes should not be taken as an index of social personality.

There are plenty of differences across social species in the dynamics of their collective behavior (Gordon, 2019), and thus each different organizational profile requires close inspection. For example, there are ant species with specialization of tasks within one single fixed foraging team, with one single nest comprising thousands of foraging teams, sometimes organized in interconnected, multi-domus nests (Reznikova, 2020). Notwithstanding the variability in social insect organization and dynamics, a rule of the thumb would be that, considering that heterogeneity of connectivity (of the network of interactions across individuals) increases with colony size (Naug, 2009), with larger colonies showing a more modularized structure (larger number of information comportments), then the larger the colony the higher should be the probability of interactions to occur between contiguous informational compartments. If, as we have shown above, the existence of reciprocal interactions (with positive and negative feedbacks) between SOSSs complies with the formal requirements for closure of constraints, the larger the colony, the more there are opportunities for the emergence of social cognition. Coupling between the dynamics of the environment and that of socially self-organized structures is not rare at all among social insect societies (Gordon, 2019), but this coupling is a sign of heteronomy. If one is interested in social, instead of individual cognition, then one is searching for autonomy, and accordingly the phenomena of interest are those that reflect the uncoupling between the flow of social information and the flow of information regarding both the external environment and the internal, social physiology.

Although none of the studied systems complies with all the formal requirements for social cognition, particularly regarding the requirement of a social self-regulation of the interactions between SOSSs, the application of the organizational approach to social systems seems to provide a more nuanced take on the issue of social personality. Some collective decisions (the ones that require the interaction between several SOSSs), performed by some species (those with positive and negative feedback systems), when measured at groups of certain (large) sizes, have the potential to be connected to a social level of personality, one that would be autonomous in relation to lower, individual levels of personality. Thus, a conceptual analysis based on a fair amount of knowledge regarding the communication structure, across and within several tasks, is required before studying social personality in any particular case.

We hope that the organizational approach herein developed helps to ground theoretically the emerging area of social personality, inspiring its application to further, related areas. For example, there have been proposals conceiving ecological communities as learning structures, with the ecological relations between species as analogs to synapses in the nervous system, and thus with individual learning, through changes in nervous system topology, as being functionally equivalent to ecosystem learning, implemented as changes in the topology of the ecological relations (Power et al., 2015). A similar proposal seems to somewhat entangle evolutionary and learning processes (Watson and Szathmáry, 2016). The application of the organizational approach would help to clarify conceptually these broad analogies. In general, while researchers in ecology are mostly interested on the biological constraints to the flux of matter and energy, our approach would instead force a focus on the cognitive constraints to the flow of information, on the psychological constraints that guide the ecological relations, helping thus to bias evolutionary processes.

## Author Contributions

All authors have written collaboratively the first version of the text. LN contributed to the review of social personality uses. NN-N contributed presenting the organizational approach. HJ contributed with the introduction, the review of social insect communication, the application of the organizational approach to social insects, and the discussion. All authors have reviewed and contributed to the final version of the text. HJ and LN contributed with the original idea, and all authors contributed to the development of the general logic of the main argument.

## Conflict of Interest

The authors declare that the research was conducted in the absence of any commercial or financial relationships that could be construed as a potential conflict of interest.

## References

[B1] Alem S. Perry C. J. Zhu X. Loukola O. J. Ingraham T. Søvik E. (2016). Associative mechanisms allow for social learning and cultural transmission of string pulling in an insect. *PLoS Biol.* 14:e1002564. 10.1371/journal.pbio.1002564 27701411 PMC5049772

[B2] Ashby W. R. (1960). *Design for a Brain: the Origin of Adaptive Behaviour.* Berlin: Springer Science & Business Media.

[B3] Beckers R. Deneubourg J. L. Goss S. (1992a). Trail laying behaviour during food recruitment in the ant *Lasius niger* (L). *Insect. Soc.* 39 59–72. 10.1007/bf01240531

[B4] Beckers R. Deneubourg J. L. Goss S. (1992b). Trails and U-turns in the selection of a path by the ant *Lasius niger*. *J. Theor. Biol.* 159 397–415. 10.1016/S0022-5193(05)80686-1

[B5] Bengston S. E. Dornhaus A. (2014). Be meek or be bold? A colony-level behavioural syndrome in ants. *Proc. R. Soc. B Biol. Sci.* 281:20140518. 10.1098/rspb.2014.0518 25100691 PMC4132670

[B6] Bengston S. E. Jandt J. M. (2014). The development of collective personality: the ontogenetic drivers of behavioral variation across groups. *Front. Ecol. Evol.* 2:81. 10.3389/fevo.2014.00081

[B7] Bergmüller R. Taborsky M. (2010). Animal personality due to social niche specialisation. *Trends Ecol. Evol.* 25 504–511. 10.1016/j.tree.2010.06.012 20638151

[B8] Blight O. Villalta I. Cerdá X. Boulay R. (2016). Personality traits are associated with colony productivity in the gypsy ant *Aphaenogaster senilis*. *Behav. Ecol. Sociobiol.* 70 2203–2209. 10.1007/s00265-016-2224-x

[B9] Bogacz R. (2007). Optimal decision-making theories: linking neurobiology with behaviour. *Trends Cogn. Sci.* 11 118–125. 10.1016/j.tics.2006.12.006 17276130

[B10] Bogacz R. Brown E. Moehlis J. Holmes P. Cohen J. D. (2006). The physics of optimal decision making: a formal analysis of models of performance in two-alternative forced-choice tasks. *Psychol. Rev.* 113:700. 10.1037/0033-295x.113.4.700 17014301

[B11] Briffa M. Greenaway J. (2011). High in situ repeatability of behaviour indicates animal personality in the beadlet anemone *Actinia equina* (Cnidaria). *PLoS One* 6:e21963. 10.1371/journal.pone.0021963 21755015 PMC3130786

[B12] Camazine S. Deneubourg J. L. Franks N. R. Sneyd J. Bonabeau E. Theraula G. (2001). *Self-organization in Biological Systems.* Princeton, NJ: Princeton University Press.

[B13] Camazine S. Visscher P. K. Finley J. Vetter R. S. (1999). House-hunting by honeybee swarms: collective decisions and individual behaviors. *Insect. Soc.* 46 348–360. 10.1007/s000400050156

[B14] Carter A. J. Feeney W. E. Marshall H. H. Cowlishaw G. Heinsohn R. (2013). Animal personality: what are behavioural ecologists measuring? *Biol. Rev.* 88 465–475. 10.1111/brv.12007 23253069

[B15] Christensen W. D. Bickhard M. H. (2002). The process dynamics of normative function. *Monist* 85 3–28. 10.5840/monist20028516

[B16] Collier J. (2006). Autonomy and process closure as the basis for functionality. *Ann. N. Y. Acad. Sci.* 901 280–291. 10.1111/j.1749-6632.2000.tb06287.x 10818579

[B17] Corr P. J. (2008). “Reinforcement sensitivity theory (RST): introduction,” in *The Reinforcement Sensitivity Theory of Personality* (Cambridge University Press), 1–43. 10.1017/CBO9780511819384.002

[B18] Corr P. J. (2010). “Individual differences in cognition: in search of a general model of behaviour control,” in *Handbook of Individual Differences in Cognition. The Springer Series on Human Exceptionality*, eds Gruszka A. Matthews G. Szymura B. (New York, NY: Springer), 3–26. 10.1007/978-1-4419-1210-7_1

[B19] Czaczkes T. J. Grüter C. Ellis L. Wood E. Ratnieks F. L. W. (2013). Ant foraging on complex trails: route learning and the role of trail pheromones in *Lasius niger*. *J. Exp. Biol.* 216 188–197. 10.1242/jeb.076570 22972897

[B20] Davidson C. J. Surette M. G. (2008). Individuality in bacteria. *Annu. Rev. Genet.* 42 253–268. 10.1146/annurev.genet.42.110807.091601 18652543

[B21] De Jaegher H. Di Paolo E. Gallagher S. (2010). Can social interaction constitute social cognition? *Trends Cogn. Sci.* 14 441–447. 10.1016/j.tics.2010.06.009 20674467

[B22] Delancey C. S. (2006). Ontology and teleofunctions: a defense and revision of the systematic account of teleological explanation. *Synthese* 150 69–98. 10.1007/s11229-004-6257-8

[B23] Dingemanse N. J. Réale D. (2013). “What is the evidence for natural selection maintaining animal personality variation?,” in *Animal Personalities: Behavior, Physiology, and Evolution*, eds Carere C. Maestripieri D. (Chicago, IL: University of Chicago Press), 201–220. 10.7208/chicago/9780226922065.003.0008

[B24] Dornhaus A. Brockmann A. Chittka L. (2003). Bumble bees alert to food with pheromone from tergal gland. *J. Comp. Physiol. A* 189 47–51. 10.1007/s00359-002-0374-y 12548429

[B25] El-Hani C. N. Nunes-Neto N. (2020). “Life on earth is not a passenger, but a driver: explaining the transition from a physicochemical to a life-constrained world from an organizational perspective,” in *Life and Evolution, History, Philosophy and Theory of the Life Sciences*, eds Baravalle L. Zaterka L. (Switzerland: Springer), 69–84. 10.1007/978-3-030-39589-6_5

[B26] Feest U. (2005). Operationism in psychology: what the debate is about, what the debate should be about. *J. Hist. Behav. Sci.* 41 131–149. 10.1002/jhbs.20079 15812819

[B27] Feinerman O. Korman A. (2017). Individual versus collective cognition in social insects. *J. Exp. Biol.* 220 73–82. 10.1242/jeb.143891 28057830 PMC5226334

[B28] Figdor C. (2018). *Pieces of Mind: The Proper Domain of Psychological Predicates.* Oxford: Oxford University Press.

[B29] Franks N. R. Mallon E. B. Bray H. E. Hamilton M. J. Mischler T. C. (2003). Strategies for choosing between alternatives with different attributes: exemplified by house-hunting ants. *Ani. Behav.* 65 215–223. 10.1006/anbe.2002.2032

[B30] Franks N. R. Pratt S. C. Mallon E. B. Britton N. F. Sumpter D. J. (2002). Information flow, opinion polling and collective intelligence in house–hunting social insects. *Philos. Trans. R. Soc. Lond. B Biol. Sci.* 357 1567–1583. 10.1098/rstb.2002.1066 12495514 PMC1693068

[B31] Franks N. R. Richardson T. (2006). Teaching in tandem-running ants. *Nature* 439:153. 10.1038/439153a 16407943

[B32] Gelblum A. Pinkoviezky I. Fonio E. Ghosh A. Gov N. Feinerman O. (2015). Ant groups optimally amplify the effect of transiently informed individuals. *Nat. Commun.* 6:7729. 26218613 10.1038/ncomms8729 PMC4525283

[B33] Gilbert S. F. Sarkar S. (2000). Embracing complexity: organicism for the 21st century. *Dev. Dyn.* 219 1–9. 10.1002/1097-0177(2000)9999:9999<::aid-dvdy1036>3.0.co;2-a 10974666

[B34] Gordon D. M. (2019). The ecology of collective behavior in ants. *Annu. Rev. Entomol.* 64 35–50. 10.1146/annurev-ento-011118-111923 30256667

[B35] Gross M. R. (1996). Alternative reproductive strategies and tactics: diversity within sexes. *Trends Ecol. Evol.* 11 92–98. 10.1016/0169-5347(96)81050-0 21237769

[B36] Heinze J. Kellner K. Seal J. (2017). “Sociality in ants,” in *Comparative Social Evolution*, eds Rubenstein D. Abbot P. (Cambridge: Cambridge University Press), 21–49. 10.1017/9781107338319.003

[B37] Holldobler B. Wilson E. O. (2009). *The Superorganism: The Beauty, Elegance, and Strangeness of Insect Societies.* WW Norton & Company: New York, NY.

[B38] Huebner B. (2013). *Macrocognition: A Theory of Distributed Minds and Collective Intentionality.* Oxford: Oxford University Press.

[B39] Ioannou C. C. Couzin I. D. James R. Croft D. P. Krause J. (2011). Social organisation and information transfer in schooling fish. *Fish Cogn. Behav.* 2 217–239. 10.1002/9781444342536.ch10

[B40] Jaffe K. Howse P. E. (1979). The mass recruitment system of the leaf cutting ant, *Atta cephalotes* (L.). *Anim. Behav.* 27 930–939. 10.1016/0003-3472(79)90031-9

[B41] Jandt J. M. Bengston S. Pinter-Wollman N. Pruitt J. N. Raine N. E. Dornhaus A. (2014). Behavioural syndromes and social insects: personality at multiple levels. *Biol. Rev.* 89 48–67. 10.1111/brv.12042 23672739

[B42] Jandt J. M. Gordon D. M. (2016). The behavioral ecology of variation in social insects. *Curr. Opin. Insect Sci.* 15 40–44. 10.1016/j.cois.2016.02.012 27436730

[B43] Japyassú H. F. Malange J. (2014). Plasticity, stereotypy, intra-individual variability and personality: handle with care. *Behav. Processes* 109 40–47. 10.1016/j.beproc.2014.09.016 25241306

[B44] Jeanson R. Deneubourg J. L. (2009). “Positive feedback, convergent collective patterns, and social transitions in arthropods,” in *Organization of Insect Societies - From Genome to Sociocomplexity*, eds Gadau J. Fewell J. (Cambridge, MA: Harvard University Press), 460–482.

[B45] Jolles J. W. Boogert N. J. Sridhar V. H. Couzin I. D. Manica A. (2017). Consistent individual differences drive collective behavior and group functioning of schooling fish. *Curr. Biol.* 27 2862–2868. 10.1016/j.cub.2017.08.004 28889975 PMC5628957

[B46] Kirchner W. H. (1993). Vibrational signals in the tremble dance of the honeybee, *Apis mellifera*. *Behav. Ecol. Sociobiol.* 33 169–172. 10.1007/bf00216597

[B47] Kolay S. Boulay R. d’Ettorre P. (2020). Regulation of ant foraging: a review of the role of information use and personality. *Front. Psychol.* 11:734. 10.3389/fpsyg.2020.00734 32425852 PMC7212395

[B48] Le Breton J. Fourcassié V. (2004). Information transfer during recruitment in the ant *Lasius niger* L. (Hymenoptera: Formicidae). *Behav. Ecol. Sociobiol.* 55 242–250. 10.1007/s00265-003-0704-2

[B49] Lynch M. Walsh B. (1998). *Genetics and Analysis of Quantitative Traits*, Vol. 1. Sunderland, MA: Sinauer, 535–557.

[B50] Mailleux A.-C. Deneubourg J.-L. Detrain C. (2003). Regulation of ants’ foraging to resource productivity. *Proc. R. Soc. B Biol. Sci.* 270 1609–1616. 10.1098/rspb.2003.2398 12908982 PMC1691411

[B51] Mallon E. B. Pratt S. C. Franks N. R. (2001). Individual and collective decision-making during nest site selection by the ant *Leptothorax albipennis*. *Behav. Ecol. Sociobiol.* 50 352–359. 10.1007/s002650100377

[B52] Marshall J. A. Bogacz R. Dornhaus A. Planqué R. Kovacs T. Franks N. R. (2009). On optimal decision-making in brains and social insect colonies. *J. R. Soc. Interf.* 6 1065–1074. 10.1098/rsif.2008.0511 19324679 PMC2827444

[B53] Marting P. R. Wcislo W. T. Pratt S. C. (2018). Colony personality and plant health in the Azteca-Cecropia mutualism. *Behav. Ecol.* 29 264–271. 10.1093/beheco/arx165 30568295 PMC6299286

[B54] Moreno A. (2018). On minimal autonomous agency: natural and artificial. *Complex Syst.* 27 289–313. 10.25088/complexsystems.27.3.289

[B55] Moreno A. Mossio M. (2015). *Biological Autonomy: A Philosophical and Theoretical Enquiry*, Vol. 12. Berlin: Springer.

[B56] Mossio M. Montévil M. Longo G. (2016). Theoretical principles for biology: organization. *Prog. Biophys. Mol. Biol.* 122 24–35. 10.1016/j.pbiomolbio.2016.07.005 27521451

[B57] Mossio M. Moreno A. (2010). Organisational closure in biological organisms. *Hist. Philos. Life Sci.* 32 269–288. 21162371

[B58] Mossio M. Saborido C. Moreno A. (2009). An organizational account of biological functions. *Br. J. Philos. Sci.* 60 813–841. 10.1093/bjps/axp036 32691291

[B59] Naug D. (2009). Structure and resilience of the social network in an insect colony as a function of colony size. *Behav. Ecol. Sociobiol.* 63 1023–1028. 10.1007/s00265-009-0721-x

[B60] Neubauer A. C. Fink A. (2010). “Neuroscientific approaches to the study of individual differences in cognition and personality,” in *Handbook of Individual Differences in Cognition: Attention, Memory, and Executive Control*, eds Gruszka A. (New York, NY: Springer), 73–85. 10.1007/978-1-4419-1210-7_5

[B61] Nunes-Neto N. Moreno A. El-Hani C. (2014). Function in ecology: an organizational approach. *Biol. Philos.* 29 123–141. 10.1007/s10539-013-9398-7

[B62] O’Donnell S. Bulova S. J. (2007). Worker connectivity: a review of the design of worker communication systems and their effects on task performance in insect societies. *Insect. Soc.* 54 203–210. 10.1007/s00040-007-0945-6

[B63] Pasquier G. Grüter C. (2016). Individual learning performance and exploratory activity are linked to colony foraging success in a mass-recruiting ant. *Behav. Ecol.* 27 1702–1709.

[B64] Planas-Sitja I. Deneubourg J. L. Gibon C. Sempo G. (2015). Group personality during collective decision-making: a multi-level approach. *Proc. R. Soc. B Biol. Sci.* 282:20142515. 10.1098/rspb.2014.2515 25652834 PMC4344149

[B65] Power D. A. Watson R. A. Szathmáry E. Mills R. Powers S. T. Doncaster C. P. (2015). What can ecosystems learn? Expanding evolutionary ecology with learning theory. *Biol. Direct* 10:69. 26643685 10.1186/s13062-015-0094-1 PMC4672551

[B66] Reznikova Z. (2017). *Studying Animal Languages Without Translation: An Insight From Ants.* Berlin: Springer International Publishing.

[B67] Reznikova Z. (2020). Spatial cognition in the context of foraging styles and information transfer in ants. *Anim. Cogn.* [Epub ahead of print]. 10.1007/s10071-020-01423-x 32840698

[B68] Reznikova Z. Ryabko B. (2011). Numerical competence in animals, with an insight from ants. *Behaviour* 148 405–434. 10.1163/000579511X568562

[B69] Robinson E. J. H. Jackson D. E. Holcombe M. Ratnieks F. L. W. (2005). No entry signal in ant foraging. *Nature* 438:442. 10.1038/438442a 16306981

[B70] Sandeman D. Tautz J. Lindauer M. (1996). Transmission of vibration across honeycombs and its detection by bee leg receptors. *J. Exp. Biol.* 199 2585–2594. 9320517 10.1242/jeb.199.12.2585

[B71] Sasaki T. Pratt S. C. (2011). Emergence of group rationality from irrational individuals. *Behav. Ecol.* 22 276–281. 10.1093/beheco/arq198 27193460

[B72] Sasaki T. Pratt S. C. (2018). The psychology of superorganisms: collective decision making by insect societies. *Annu. Rev. Entomol.* 63 259–275. 10.1146/annurev-ento-020117-043249 28977775

[B73] Scharf I. Modlmeier A. P. Fries S. Tirard C. Foitzik S. (2012). Characterizing the collective personality of ant societies: aggressive colonies do not abandon their home. *PLoS One* 7:e33314. 10.1371/journal.pone.0033314 22457751 PMC3310061

[B74] Schlosser G. (1998). Self-re-production and functionality: a systems-theoretical approach to teleological explanation. *Synthese* 116 303–354.

[B75] Seeley T. D. (1995). *The Wisdom of the Hive: The Social Physiology of the Honey Bee Hives.* Cambridge, MA: Harvard University Press.

[B76] Seeley T. D. Buhrman S. (1999). Group decision-making in swarms of honeybees. *Behav. Ecol. Sociobiol.* 45 19–31.

[B77] Seeley T. D. Buhrman S. C. (2001). Nest-site selection in honeybees: how well do swarms implement the ‘best-of-N’ decision rule? *Behav. Ecol. Sociobiol.* 49 416–427. 10.1007/s002650000299

[B78] Seeley T. D. Visscher P. K. Schlegel T. Hogan P. M. Franks N. R. Marshall J. A. (2012). Stop signals provide cross inhibition in collective decision-making by honeybee swarms. *Science* 335 108–111. 10.1126/science.1210361 22157081

[B79] Shettleworth S. J. (2010). *Cognition, Evolution, and Behavior.* Oxford: Oxford University Press.

[B80] Sih A. Bell A. M. Johnson J. C. Ziemba R. E. (2004). Behavioral syndromes: an integrative overview. *Q. Rev. Biol.* 79 241–277. 10.1086/422893 15529965

[B81] Solé R. Moses M. Forrest S. (2019). Liquid brains, solid brains. *Philos. Trans. R. Soc. B* 374 1–6. 10.1098/rstb.2019.0040 31006374 PMC6553592

[B82] Sumpter D. J. (2010). *Collective Animal Behavior.* Princeton, NY: Princeton University Press.

[B83] Sumpter D. J. T. Beekman M. (2003). From nonlinearity to optimality: pheromone trail foraging by ants. *Anim. Behav.* 66 273–280. 10.1006/anbe.2003.2224

[B84] Theraulaz G. Bonabeau E. (1999). A brief history of stigmergy. *Artif. Life* 5 97–116. 10.1162/106454699568700 10633572

[B85] Thom C. Gilley D. C. Hooper J. Esch H. E. (2007). The scent of the waggle dance. *PLoS Biol.* 5:e228. 10.1371/journal.pbio.0050228 17713987 PMC1994260

[B86] Thom C. Gilley D. C. Tautz J. (2003). Worker piping in honey bees (*Apis mellifera*): the behavior of piping nectar foragers. *Behav. Ecol. Sociobiol.* 53 199–205. 10.1007/s00265-002-0567-y

[B87] van Oers K. De Jong G. Van Noordwijk A. J. Kempenaers B. Drent P. J. (2005). Contribution of genetics to the study of animal personalities: a review of case studies. *Behaviour* 142 1185–1206. 10.1163/156853905774539364

[B88] Visscher P. K. Camazine S. (1999). Collective decisions and cognition in bees. *Nature* 397:400. 10.1038/17047 29667972

[B89] Watson R. A. Szathmáry E. (2016). How can evolution learn? *Trends Ecol. Evol.* 31 147–157. 10.1016/j.tree.2015.11.009 26705684

[B90] Wcislo W. Fewell J. (2017). “Sociality in bees,” in *Comparative Social Evolution*, ed. Rubenstein D. R. (Cambridge: Cambridge University Press), 50–83. 10.1017/9781107338319.004

[B91] Widemo F. (1998). Alternative reproductive strategies in the ruff, Philomachuspugnax: a mixed ESS? *Anim. Behav.* 56 329–336. 10.1006/anbe.1998.0792 9787023

[B92] Wilson E. O. (1971). *The Insect Societies.* Cambridge, MA: Harvard University Press.

[B93] Wolfe C. T. (2010). Do organisms have an ontological status? *Hist. Philos. Life Sci.* 32 195–231. 21162368

[B94] Wray M. K. Mattila H. R. Seeley T. D. (2011). Collective personalities in honeybee colonies are linked to colony fitness. *Anim. Behav.* 81 559–568. 10.1016/j.anbehav.2010.11.027

[B95] Wright C. M. Lichtenstein J. L. Doering G. N. Pretorius J. Meunier J. Pruitt J. N. (2019). Collective personalities: present knowledge and new frontiers. *Behav. Ecol. Sociobiol.* 73:31.

[B96] Yan H. Opachaloemphan C. Mancini G. Yang H. Gallitto M. Mlejnek J. (2017). An engineered orco mutation produces aberrant social behavior and defective neural development in ants. *Cell* 170 736–747. 10.1016/j.cell.2017.06.051 28802043 PMC5587193

